# Translation and validation of a Hebrew version of the Western Ontario Shoulder Instability index

**DOI:** 10.1186/s13018-019-1289-4

**Published:** 2019-08-01

**Authors:** Uri Gottlieb, Shmuel Springer

**Affiliations:** 1 Israel Defense Forces, Medical Corps, Ramat-Gan, Israel; 2 Department of Physical Therapy, Ariel University, 40700 Ariel, Israel

**Keywords:** Evaluation, Shoulder instability, Quality of life, Self-administered questionnaire, Patient-reported outcome measures

## Abstract

**Background:**

The Western Ontario Shoulder Instability index (WOSI) is a questionnaire designed to measure health-related quality of life in patients with shoulder instability. The aim of the current study was to translate the WOSI into Hebrew and assess its psychometric properties.

**Methods:**

The WOSI was translated into Hebrew according to World Health Organization guidelines. Twenty-five patients completed the WOSI and the Disabilities of Arm, Shoulder, and Hand (DASH) questionnaire 2 weeks and 2 months after surgical shoulder stabilization. Internal consistency (Cronbach’s α), criterion validity (Pearson’s correlation coefficient with DASH), responsiveness, and floor and ceiling effects were assessed.

**Results:**

Cronbach’s α was 0.88–0.95 for total WOSI (range 0.68–0.95 for different sections). Strong correlation with DASH score (*r* = 0.76–0.84) indicated good criterion validity. Changes between baseline and follow-up for WOSI and DASH scores were moderately correlated (*r* = 0.68), suggesting moderate responsiveness. Some items demonstrated floor and ceiling effects, especially at baseline, but no floor or ceiling effects were observed for total WOSI or for the WOSI sections.

**Conclusions:**

The results of the current study demonstrate that the Hebrew version of the WOSI is a valid instrument that can be used to assess disability in patients with shoulder instability. Additional studies are warranted to assess its psychometric properties among various subpopulations.

**Trial registration:**

The study was pre-registered at the ClinicalTrials.gov website, registration number NCT02978365.

## Introduction

Anterior shoulder dislocations are the primary cause of shoulder instability (SI), a condition that refers to the inability to maintain the humeral head in the glenoid fossa [1]. The incidence of primary shoulder dislocation is 8.2 to 23.9 per 100,000 person-years, and its estimated prevalence is 1.7% [2, 3]. About two thirds of shoulder dislocations will evolve into SI within five years [4]. Recurrent dislocations are also common, affecting 59–96% of youth (< 20 years old) and 40–74% of adults (20–40 years old) after primary dislocation [5]. In patients with SI, the ability to participate in sports-related activities is often inhibited, resulting in decreased quality of life [4]. Due to high recurrence rates and unsatisfactory outcomes after non-operative rehabilitation [6, 7], more than 60% of patients choose surgical treatment [8].

Results of either conservative or surgical treatment should be evaluated objectively and subjectively. Objective evaluation includes measurement of range of motion and rotator cuff strength, provocative physical examination maneuvers, and rate of re-dislocations [9]. Subjective measures include questionnaires, referred to as patient-reported outcome measures (PROMs), that assess patient function and satisfaction [10]. A PROM commonly used to evaluate upper extremity function is the Disabilities of Arm, Shoulder, and Hand questionnaire (DASH) [11]**.** The DASH can detect and differentiate small and large changes in disability over time after surgery in patients with a variety of upper-extremity musculoskeletal disorders [12–14]**.**

The Western Ontario Shoulder Instability index (WOSI) is a questionnaire designed to measure health-related quality of life in patients with SI [15]. This instrument consists of four subscales: (1) physical symptoms and pain; (2) sport, recreation, and work function; (3) lifestyle and social functioning; and (4) emotional well-being. The 21 items are scored using a visual analog scale, measuring 100 mm horizontally, placed under each question. The best possible score indicating the highest possible shoulder-related quality of life is 0, and 100 is the worst possible. It has been thoroughly evaluated for reliability, validity, and sensitivity for change, both pre- and post-operatively [12, 16–18]. The WOSI has been translated and culturally adapted into several languages [19–23].

Translation and validation of PROMs allow comparison of national and international study results [24, 25]. In addition, it allows patient evaluation using internationally accepted instruments. Recently, the Israeli Physical Therapy Society has encouraged clinicians and researchers to translate and validate PROMs into Hebrew, for clinical use. Therefore, the aim of this study was to translate and validate a Hebrew version of the WOSI (H-WOSI).

## Methods

The translation process was conducted according to the World Health Organization (WHO) requirements for translation and cultural adaptation [26, 27] and included the following: (1) forward translation into Hebrew by a native Hebrew speaker, (2) an experts’ panel to identify and correct unclear expressions, (3) a back translation to English by a native English speaker, (4) pre-testing and interviewing five patients, and (5) accepting the final version. The Hebrew translation was done by a physical therapist that studied and worked in England for several years. The experts’ committee included three physical therapists that are very familiar with musculoskeletal injuries, as well as with research and outcome measures. The experts’ panel debated the precise words and expressions used to describe different types of pain (i.e., pain, aching, throbbing). Other changes were made to make the structure of the questions understandable for Hebrew speakers. The back translation was done by a native English speaker who does not have any medical training. The pre-testing and interview were completed with five patients who were an average of 5 (±2.8) weeks after surgical repair of SI and did not participate in further data analysis. These patients were asked to explain in their own words each item in the questionnaire, to assure comprehension. Several issues were raised by the participants and were discussed by the experts’ panel. After correcting these issues, the final version was obtained and is available as supplementary data.

Validation of the final version was conducted on 25 patients who participated in a larger study that has not been published yet. The study was approved by the IDF Medical Corps Helsinki Committee (approval number 1702-2016) and was registered at the ClinicalTrials.gov website (registration number NCT02978365). Written informed consent was obtained from all eligible patients who were recruited. Participants were male soldiers 18–30 years old who attended the military physical therapy clinic in Tzrifin from March 2017 to March 2018, after undergoing arthroscopic repair of SI.

Participants completed the H-WOSI and a Hebrew version of DASH (H-DASH) at 0–2 and 7–8 weeks post-surgery. The DASH was chosen as it was previously used to validate the WOSI in several languages [10, 15, 19, 21]. The cross-cultural adaptation of the H-DASH was previously performed by Ziv et. al and is available at the official DASH website [28]. It has been used in clinical and research settings [29].

## Data analysis

Participants’ characteristics and outcome measures are presented using descriptive statistics. Continuous variables are described as mean, standard deviation (SD), 95% CI, and range. Categorical variables are displayed as number of participants and percentage for each category.

Internal consistency, which reflects the extent to which the questionnaire items are intercorrelated or whether they measure the same construct consistently, was measured by calculating Cronbach’s α for each section of the H-WOSI, separately. Consistency was concluded when Cronbach’s α reached a minimum value of 0.7 [30].

Criterion validity refers to the extent to which scores on a particular instrument relate to a gold standard. This measure was assessed by calculating Pearson’s correlation coefficient between the H-WOSI and the H-DASH at the two measurement intervals (baseline and follow-up). Criterion validity was defined when the correlation coefficient *r* > 0.7 [30].

Responsiveness is defined as the ability of a questionnaire to detect clinically important changes over time. To assess responsiveness of the H-WOSI, a correlation coefficient was calculated between the baseline and follow-up change in the H-WOSI, as compared to the change in H-DASH. Changes were calculated as percentage of baseline score. Standardized response mean (SRM) was also calculated for both measures.

Floor (worst possible) and ceiling (best possible) effects were defined as the bottommost and topmost scores for each item, respectively. A floor or a ceiling effect was concluded when > 15% of respondents achieved the lowest or highest possible score [30].

IBM Statistical Package for the Social Sciences (SPSS) v.23.0 was used for all analyses.

## Results

Twenty-five patients were included in final analysis. Baseline characteristics of participants are described in Table 1. The results of each questionnaire are shown in Table 2.

**Table 1:** Baseline characteristics of participants

Participant characteristics (*n* = 25)	Mean (SD)	Range
Age (years)	20.7 (1.2)	19–24
Time from surgery to baseline (days)	10.7 (5.1)	3–26
Time from surgery to follow-up (days)	56.1 (5.5)	47–71
	Number of participants (%)	
Dominant side (right/left)	20 (80%)/5 (20%)	
Operated side (right/left)	17 (68%)/8 (32%)	
Dominant side operated (yes/no)	14 (56%)/11 (44%)

**Table 2:** Baseline and follow-up outcome measures and comparisons

	Baseline			Follow-up		
	Mean (SD)	95% CI	Range	Mean (SD)	95% CI	Range
H-DASH	57.1 (19.1)	49.6–64.6	9.1–90.8	33.3 (20.0)	25.4–41.2	0.0–80.0
Total H-WOSI	71.2 (14.9)	65.3–77.1	29.5–99.3	49.8 (21.7)	41.3–58.3	0.67–81.2
Physical	64.1 (17.1)	57.4–70.8	25.9–98.6	41.9 (21.0)	33.7–50.2	0.2–71.3
Sports	87.7 (16.3)	81.3–94.1	40.5–100.0	61.9 (27.6)	51.1–72.8	1.0–100.0
Lifestyle	80.5 (17.2)	73.8–87.3	34.7–100.0	59.5 (25.2)	49.7–69.5	1.5–94.0
Emotional	60.3 (24.7)	50.6–69.9	19.7–100.0	46.7 (28.0)	35.8–57.8	0.67–100.0

*DASH* Disability of Arm, Shoulder, and Hand, *WOSI* Western Ontario Shoulder Instability index

Excellent internal consistency was found for the H-WOSI, both at baseline and at follow-up. No items were found to significantly increase consistency of the H-WOSI, if removed. Nevertheless, when assessing internal consistency of the separate sections, the lifestyle and emotional sections at baseline did not reach the minimum value of 0.7 (Cronbach’s α = 0.698 and 0.688, respectively). This information is described in Table 3.

**Table 3:** Internal consistency of the H-WOSI

	Number of items	Cronbach’s α	
		Baseline	Follow-up
Total H-WOSI	21	0.885	0.952
Physical	10	0.786	0.912
Sports	4	0.788	0.883
Lifestyle	4	0.698	0.812
Emotional	3	0.688	0.782

*H-WOSI* Hebrew version of the Western Ontario Shoulder Instability index

The H-WOSI and H-DASH scores indicated a strong correlation both at baseline (*r* = 0.768, *p* < 0.001) and at follow-up (*r* = 0.849, *p* < 0.001).

The changes in H-WOSI and H-DASH from baseline to follow-up were moderately correlated (*r* = 0.689, *p* < 0.001), suggesting fair responsiveness. The SRM for both measures was large, with SRM = 1.29 for H-WOSI and SRM = 1.5 for H-DASH.

Only one subject reached the overall ceiling score at baseline, and one at follow-up. When evaluating subscales separately, ceiling effect was found at baseline for the sports, lifestyle, and emotional sections. Furthermore, 18/21 and 2/21 items demonstrated a ceiling and floor effect at baseline, respectively. At follow-up, ceiling effect was found in 6/21 items, and floor effect for 2/21 items (Table 4).

**Table 4:** Number of subjects (%) who displayed floor or ceiling effect in each subscale

		Physical	Sports	Lifestyle	Emotional	Total
Baseline	Floor effect	0 (0%)	0 (0%)	0 (0%)	0 (0%)	0 (0%)
	Ceiling effect	0 (0%)	8 (32%)	4 (16%)	4 (16%)	1 (4%)
Follow-up	Floor effect	1 (4%)	1 (4%)	0 (0%)	1 (4%)	0 (0%)
	Ceiling effect	0 (0%)	2 (8%)	0 (0%)	1 (4%)	1 (4%)

*H-WOSI* Hebrew version of the Western Ontario Shoulder Instability index

## Discussion

Translation and validation of measurement tools are important assets for local research and international collaborative study, as well as treatment optimization. The translation and validation of H-WOSI resulted in a valid instrument to measure shoulder-related quality of life in patients with SI.

Patients who underwent surgical shoulder stabilization participated in the current study. Disability immediately after surgery is temporary and is mainly caused by the surgery itself, rather than the condition that led to it. Two months after surgery, a considerable reduction in disability is expected, but not complete return to athletic activity. The WOSI was designed to assess outcomes in all patients with SI, including pre-operatively and post-operatively. The original version of the WOSI was validated on patients at 2 weeks and 3 months after shoulder stabilization, similarly to the current study [15].

Internal consistency of the H-WOSI was excellent at baseline (Cronbach’s α = 0.885) and at follow-up (Cronbach’s α = 0.952). Similar results were previously reported for several translations of the WOSI [10, 19, 22]. van der Linde et al. [10] reported Cronbach’s α = 0.96 for total WOSI score in 138 patients with SI. Similarly, Salomonsson et al. [22], who examined patients before and 6 months after surgery, reported Cronbach’s α = 0.89–0.95. While high internal consistency was evident in all subsections of the WOSI at follow-up, the lifestyle and emotional subscales at baseline demonstrated lower internal consistency. This finding is partly in agreement with a relatively low internal consistency in the lifestyle section reported by Salomonsson et al [22]. As suggested by van der Linde et al. [10], some sections of the WOSI may lack face validity. Although questions regarding fear of falling or sleeping difficulties are included in the lifestyle section, they represent emotional aspects or physical symptoms. Therefore, it was suggested that the subscales should be used with caution [10].

Criterion validity of the H-WOSI was evaluated by comparing it to the H-DASH score, as done in previous studies that translated and validated this instrument [10, 19, 21, 23]. The current findings demonstrate that the H-WOSI has excellent criterion validity immediately after surgery (*r* = 0.768) and at short-term follow-up (*r* = 0.849). These results are consistent with previous reports that showed strong correlations between WOSI and DASH scores. van der Linde et al. [10] evaluated the Dutch version of the WOSI and reported a correlation coefficient of *r* = 0.81 with DASH scores in 138 patients with SI. Similarly, Cacchio et al. [19] reported a correlation coefficient of *r* = 0.79 between the Italian version of the WOSI and DASH, in a population of 64 patients with SI. The strong, yet not perfect, relationship between the WOSI and DASH confirms that the WOSI is a valid tool to evaluate shoulder function. However, the finding that the correlation is not absolute emphasizes the differences between the two instruments. This may be attributed to the specific characteristics of the WOSI in evaluating subjects with SI. The DASH only evaluates functional tasks and symptoms, while the WOSI also includes items that assess emotions and lifestyle. Moreover, several items in the DASH are often not relevant to patients with SI, such as writing, opening a jar, or turning a key.

Although reliability was not directly assessed, the strength of the agreement between the H-WOSI and H-DASH on the same patients at two post-operative intervals can provide some indication regarding reliability. Furthermore, the changes in H-WOSI and H-DASH from baseline to follow-up were moderately correlated. This means that subjects who improved according to the H-DASH also improved according to H-WOSI, and that the H-WOSI was sensitive for detecting these changes. Therefore, it is suggested that the translated H-WOSI has acceptable levels of responsiveness.

Both floor and ceiling effects were identified for several items, with a very large ceiling effect at baseline. More than 20% of patients demonstrated a ceiling effect for 15 of 21 items at baseline. This is expected, as certain items refer to actions that the participants were not allowed to perform at that stage of rehabilitation. On the other hand, when floor and ceiling effects were measured per section and not per item, only the sports section at baseline demonstrated a ceiling effect. Therefore, it is suggested that the H-WOSI has acceptable floor and ceiling effects, although caution should be used when assessing patients immediately after surgery.

This study had several limitations. It was conducted on a relatively small sample size of 25 patients, which is substantially smaller than the recommended sample size of 50 for validation studies [27, 30]. Yet, the process of translation and adaption of an instrument may be less demanding, as suggested by the WHO guidelines for translating and adapting instruments [26].

Test-retest reliability was not directly assessed. Therefore, several psychometric measures, such as standard error of the measurement or the minimal detectable change, could not be calculated. Nevertheless, it is unlikely that the Hebrew translation would result in any differences regarding these features.

Despite these limitations, we suggest that the H-WOSI can be adopted for clinical and laboratory use, as the psychometric properties measured in the current study were very similar to those found in previous translation and validation studies of the WOSI.

In summary, based on the results of the current study, the H-WOSI seems to be a valid instrument that can be used to assess disability in Hebrew-speaking patients with SI. The utilization of the H-WOSI in clinical practice and research should be encouraged in order to obtain a better perspective of the patient’s functional status, as well as to measure progress. Furthermore, the specific characteristics of the WOSI compared to traditional instruments make it preferable tool for evaluating subjects with SI. Further studies are warranted to assess psychometric properties of the WOSI among various subpopulations.

## Data Availability

The datasets used and/or analyzed during the current study are available from the corresponding author on reasonable request.

## Abbreviations

DASH/H-DASH: Disabilities of Arm, Shoulder, and Hand/Hebrew version
PROM: Patient-reported outcome measure
SD: Standard deviation
SI: Shoulder instability
SPSS: Statistical Package for the Social Sciences
SRM: Standardized response mean
WHO: World Health Organization
WOSI/H-WOSI: Western Ontario Shoulder Instability index/Hebrew version
